# Supplementary material for: Apolipoproteins—New Biomarkers of Overweight and Obesity among Childhood Acute Lymphoblastic Leukemia Survivors?
Source: Int J Mol Sci. 2022 Sep 13;23(18):10634. doi: 10.3390/ijms231810634 (PMC9505294; doi:10.3390/ijms231810634)
Supplement: Supplementary file 1 [file ijms-23-10634-s001.zip › ijms-1885201-supplementary.pdf]

Table S1. Characteristics of patients with acute lymphoblastic leukemia (ALL) according to gender. Data are presented as mean ± standard deviation (SD).

	Total *n*=58	Females *n*=27	Males *n*=31	*p*-value (females vs. males)
TAG (mg/dl)	97.65 ± 48.18	95.77 ± 51.94	100.50 ± 43.17	0.543
Apo-A1 (mg/dl)	259.55 ± 125.56	284.74 ± 131.92	231.56 ± 114.02	0.111
Apo-A2 (mg/dl)	24.75 ± 6.39	25.09 ± 6.20	24.34 ± 6.70	0.877
Apo-B (mg/dl)	61.15 ± 14.86	60.43 ± 14.71	61.99 ± 15.29	0.700
Apo-C1 (mg/dl)	20.50 ± 6.62	20.37 ± 7.22	20.65 ± 6.00	0.878
Apo-C3 (mg/dl)	5.33 ± 1.95	5.47 ± 1.97	5.18 ± 1.94	0.596
Apo-D (mg/dl)	3.61 ± 0.67	3.71 ± 0.58	3.51 ± 0.75	0.411
Apo-E (mg/dl)	2.71 ± 1.09	2.69 ± 1.05	2.73 ± 1.15	0.890
Apo-H (mg/dl)	41.56 ± 10.44	43.02 ± 9.35	39.93 ± 11.49	0.544
Apo-J (mg/dl)	11.10 ± 3.50	11.36 ± 2.82	10.82 ± 4.17	0.482
CRP (mg/dl)	0.85 ± 0.85	0.82 ± 0.70	0.89 ± 1.00	0.785

Table S2. Characteristics of patients with acute lymphoblastic leukemia (ALL) according to radiotherapy. Data are presented as mean ± standard deviation (SD).

	Radiotherapy *n*=9	Non-radiotherapy *n*=49	*p*-value
TAG (mg/dl)	131.00 ± 58.08	90.03 ± 43.03	0.059
Apo-A1 (mg/dl)	261.03± 140.56	251.88 ± 114.88	0.834
Apo-A2 (mg/dl)	24.84 ± 6.96	24.27 ± 6.08	0.988
Apo-B (mg/dl)	63.42 ± 20.89	60.16 ± 13.70	0.555
Apo-C1 (mg/dl)	21.27 ± 5.26	19.86 ± 6.58	0.547
Apo-C3 (mg/dl)	6.06 ± 2.28	5.03 ± 1.71	0.127
Apo-D (mg/dl)	3.76 ± 0.57	3.53 ± 0.63	0.409
Apo-E (mg/dl)	3.30 ± 1.76	2.56 ± 0.88	0.232
Apo-H (mg/dl)	43.88 ± 6.48	40.36 ± 10.42	0.283
Apo-J (mg/dl)	12.52 ± 3.96	10.61 ± 3.11	0.114
CRP (mg/dl)	1.26 ± 1.62	0.78 ± 0.61	0.381

Table S3. Spearman rank correlation of apolipoprotein in patients with acute lymphoblastic leukemia (ALL) depending on selected basic parameters.

Variable		Spearman r	*p*-value	95% confidence interval
BMI (kg/m^2)	Apo-A1	0.22	0.282	-0.19 – 0.57
	Apo-A2	0.30	0.145	-0.12 – 0.63
	Apo-B	0.31	0.117	-0.09 – 0.63
	Apo-C1	0.26	0.203	-0.15 – 0.59
	Apo-C3	0.60	0.001	0.27 – 0.81
	Apo-D	0.56	0.003	0.21 – 0.78
	Apo-E	0.37	0.063	-0.03 – 0.67
	Apo-H	0.49	0.010	0.12 – 0.74
	Apo-J	0.41	0.037	0.01 – 0.69
	CRP	0.47	0.016	0.09 – 0.73
WHR	Apo-A1	-0.12	0.570	-0.50 – 0.30
	Apo-A2	0.11	0.602	-0.32 – 0.50
	Apo-B	0.25	0.219	-0.17 – 0.60
	Apo-C1	0.16	0.437	-0.26 – 0.53
	Apo-C3	0.04	0.852	-0.37 – 0.44
	Apo-D	0.16	0.434	-0.26 – 0.53
	Apo-E	0.06	0.779	-0.35 – 0.45
	Apo-H	0.07	0.753	-0.35 – 0.46
	Apo-J	0.01	0.950	-0.39 – 0.42
	CRP	0.38	0.059	-0.03 – 0.68
Age at diagnosis (years)	Apo-A1	-0.32	0.104	-0.64 – 0.08
	Apo-A2	-0.16	0.443	-0.53 – 0.26
	Apo-B	0.07	0.717	-0.33 – 0.46
	Apo-C1	0.08	0.699	-0.33 – 0.46
	Apo-C3	0.16	0.444	-0.26 – 0.52
	Apo-D	0.23	0.256	-0.18 – 0.57
	Apo-E	-0.07	0.744	-0.45 – 0.34
	Apo-H	0.17	0.415	-0.25 – 0.53

	Apo-J	-0.07	0.736	-0.45 – 0.34
	CRP	0.32	0.104	-0.08 – 0.64
SBP (mmHg)	Apo-A1	-0.06	0.757	-0.45 – 0.34
	Apo-A2	0.08	0.708	-0.34 – 0.47
	Apo-B	0.22	0.276	-0.19 – 0.57
	Apo-C1	0.32	0.114	-0.09 – 0.63
	Apo-C3	0.46	0.018	0.08 – 0.72
	Apo-D	0.35	0.077	-0.05 – 0.66
	Apo-E	0.32	0.107	-0.08 – 0.64
	Apo-H	0.36	0.071	-0.04 – 0.66
	Apo-J	0.22	0.277	-0.19 – 0.57
	CRP	0.32	0.110	-0.09 – 0.64
DBP (mmHg)	Apo-A1	-0.13	0.536	-0.50 – 0.28
	Apo-A2	0.23	0.263	-0.19 – 0.58
	Apo-B	0.35	0.077	-0.05 – 0.66
	Apo-C1	0.46	0.018	0.08 – 0.72
	Apo-C3	0.35	0.077	-0.05 – 0.66
	Apo-D	0.34	0.087	-0.06 – 0.65
	Apo-E	0.19	0.343	-0.22 – 0.54
	Apo-H	0.36	0.074	-0.05 – 0.66
	Apo-J	0.08	0.683	-0.32 – 0.47
	CRP	-0.04	0.838	-0.43 – 0.36
